# Phytoconstituents, antioxidant and enzyme inhibition activities of coffee beans through widely targeted metabolomes and *in vitro* arrays

**DOI:** 10.1016/j.fochx.2025.103280

**Published:** 2025-11-10

**Authors:** Yuan Wang, Zizheng Huang, Hongyan He, Xiandong Zhou, Dingshan Yang, Peng Shu, Xueqing Chen, Jinhuan Cheng, Yao Ma

**Affiliations:** a HBN Research Institute and Biological Laboratory, Shenzhen Hujia Technology Co., Ltd., Shenzhen 518000, China; b School of Life Sciences, Zhengzhou University, Zhengzhou 450001, China; c Henan Funiu Mountain Biological and Ecological Environment Observatory, Zhengzhou 450001, China; d Yunnan Academy of Agriculture Engineering, Kunming 650200, China

**Keywords:** Coffee, Bioactivity, Metabolomics, Chemometrics, Potential bioactive compounds

## Abstract

Coffee has transcended from a beverage into a functional food, with its efficacy heavily influenced by germplasm diversity. We systematically analyzed 37 coffee accessions, revealing substantial diversity in morphological characteristics, crude extract yield, antioxidant capacity, and enzyme inhibition. Widely targeted metabolomics on three accessions exhibiting distinct bioactivities identified 2,933 compounds. The predominant classes, with representative metabolites, include 404 terpenoids (e.g., cafestol and kahweol), 362 flavonoids (e.g., tangeretin and rutin), and 340 phenolic acids (e.g., chlorogenate and cryptochlorogenic acid). Flavonoids and organic acids were the main differential metabolites across the three accessions and were enriched in relevant metabolic pathways. K-means and correlation analysis confirmed that flavonoids and organic acids, particularly syringetin-3-O-glucoside and chlorogenic acid, were key bioactivity drivers. Our study constructs a comprehensive metabolite and bioactive database for the rational selection of coffee accessions in the development of functional foods, dietary supplements, and cosmeceuticals with targeted health benefits.

## Highlights

• Coffee beans showed multifunctional activities.
• Widely targeted metabolomics profiled three coffees with distinct bioactivities.
• Flavonoids and organic acids drive the observed bioactivity differences.

## 1 Introduction

Coffee, one of the most extensively traded stimulant beverages, has evolved beyond its traditional role to emerge as a scientifically validated nutraceutical (Grzelczyk et al., 2024; Hong et al., 2025). Advances in phytochemical profiling have systematically elucidated the complex metabolomes in coffee beans, identifying structurally diverse bioactive constituents, including flavonoids, phenolic acids, alkaloids, and terpenoids (Bichlmaier et al., 2025; Hong et al., 2025; Pimenta et al., 2025; Santanatoglia et al., 2024; Wu et al., 2025). Among the bioactive constituents identified, hydroxycinnamic acid and its derivatives (quinic acid esters with caffeoyl or feruloyl groups) demonstrate pronounced pharmacological effects (Grzelczyk et al., 2024; Santanatoglia et al., 2024). Extensive research has confirmed that chlorogenic acids possess a range of significant biological properties, including antioxidant, antidiabetic, neuroprotective, and anti-inflammatory activities (Han et al., 2024; Huang et al., 2023; Liu et al., 2024). The compelling bioactivity profile of bioactive constituents in coffee positions it as a potential candidate for innovative cosmeceutical applications targeting oxidative stress, hyperpigmentation, and inflammatory dermatoses.

Nevertheless, the translation of coffee bioactivity into effective functional cosmeceutical formulations is impeded by several critical challenges. Firstly, pronounced metabolic divergence among coffee cultivars gives rise to highly inconsistent biological efficacy (Alvarenga et al., 2025; Grzelczyk et al., 2024; Pimenta et al., 2025; Santanatoglia et al., 2024; Wu et al., 2025). Secondly, contemporary cosmeceutical evaluation paradigms remain predominantly reliant on single-target *in vitro* assays (e.g. radical scavenging capacity or tyrosinase inhibition activity), neglecting the multifactorial complexity of skin physiology. An exclusive focus on tyrosinase inhibition fails to consider the potential interplay between pigmentation control and dermal matrix integrity, which is mediated through the activation of hyaluronan synthase or the regulation of metalloproteinase (Lu et al., 2024). Furthermore, the field persistently prioritises isolated compound analyses over holistic evaluations of phytocomplex synergism, inadequately capturing critical functional synergies of plant bioactive constituents (Liu et al., 2024; Lu et al., 2022). It is imperative to acknowledge the absence of curated metabolic databases linking cultivar-specific biomarkers to quantifiable skincare endpoints impedes the translation of rich bioactive constituents found in coffee into multifunctional cosmeceutical formulations (Alvarenga et al., 2025; Wu et al., 2025).

Consequently, a set of 37 diverse *C. arabica* accessions was selected for this study to ensure a comprehensive representation of the species' metabolic and bioactivity diversity, enabling robust statistical analysis and the identification of key efficacy-driving compounds. The antioxidant capacity was evaluated using 2,2-diphenyl-1-picrylhydrazyl (DPPH) and diammonium 2,2'-azino-bisbis (3-ethylbenzothiazoline-6-sulfonate) (ABTS^+^•) radical scavenging combined with ferric reducing antioxidant power (FRAP) and reducing power (RP). Secondly, dual-substrate tyrosinase inhibition kinetics using both L-tyrosine and L-3,4-dihydroxyphenylalanine (L-DOPA) were investigated. Thirdly, the anti-inflammatory assessment was conducted through the hemolysis and hyaluronidase inhibition capacities. Subsequently, a widely targeted metabolomics approach was applied to detect metabolic profiles of three distinct coffee accessions, with the aim of establishing metabolite-activity relationships. This strategy has enabled the elucidation of the metabolic determinants underpinning biological efficacy, as well as the development of a novel Genotype-Selection algorithm for targeted skincare applications. The present study resolves the synergistic interplay between antioxidant networks, tyrosinase inhibition, and anti-inflammatory pathways, facilitating a paradigm shift from empirical formulation towards metabolome-guided precision cosmeceutical engineering.

## 2 Materials and methods

### 2.1 Plant material collection and treatment

The mature fruits of 37 coffee accessions were harvested at Aini Coffee Estate in Yunnan province, China (**Table S1**). The fruits exhibiting uniform size, intact pericarp, and absence of visual defects (disease, pest damage, mechanical injury) were selected, and relevant data (e.g., harvest date, location, cultivar) were recorded. The samples were then transferred directly to the laboratory. The fruits were meticulously washed with deionized water and blot-dried. The samples were divided into two parts: one subset was reserved intact for immediate morphometric analysis, and the remaining fruits were freeze-dried and pulverized to a homogeneous powder. The resulting powder was stored at -80 °C for all subsequent biochemical and metabolomic analyses as needed.

### 2.2 Fruit morphometric characteristics

A randomised sampling involving ten fruits per coffee accession was employed for the morphometric evaluation. The fruit fresh weight was determined using an analytical balance (Ohaus Explorer, readability 0.0001 g). The fruit shape index (FSI) was derived from the longitudinal diameter (maximum apex-to-stem axis) and the transverse diameter (equatorial cross-sectional), which were measured in triplicate per fruit using a digital vernier caliper (Mitutoyo 500-196-30, resolution 0.01 mm).

### 2.3 *In vitro* biological activities

Beyond the scope of morphological and physical characteristics, a systematic evaluation was conducted of the bioactivities of extracts from all 37 coffee accessions. The assessment encompassed four distinct functions: antioxidant, tyrosinase inhibitory, anti-haemolysis, and hyaluronidase inhibitory profiles. All experiments were conducted with three biological replicates for each accession. The initial concentration of each extract was set at 5.0 mg/mL, and assays were performed according to established protocols with slight modifications as required.

#### 2.3.1 Measurements of antioxidant activities

The *in vitro* antioxidant capacities were comprehensively evaluated through four complementary analytical approaches: the DPPH and ABTS^+^• radical scavenging assays, FRAP and RP assessment. These evaluations were performed in accordance with the established protocol (Ma et al., 2019). Reaction mixtures containing equivalent volumes of methanol served as solvent blanks, while equivalent volumes of ascorbic acid were employed as the positive control throughout the study.

#### 2.3.2 Tyrosinase inhibitory activity

The tyrosinase inhibitory activity of the samples was determined through a spectrophotometric method, following the optimised enzymatic assay protocol (Zhao et al., 2025). Different concentration samples (50 μL) were combined with tyrosinase solution (200 U/mL, 50 μL) in phosphate buffer (PBS, 0.1 M, pH 6.8), and then incubated at 25 ^o^C for 15 minutes in darkness. Subsequently, the reaction mixture was added to a solution of L-DOPA or tyrosine solution (100 μL of 2 mM) for a period of 15 minutes. Absorbance values were measured at 475 nm. The PBS at equivalent volumes served as solvent blanks, while kojic acid was employed as a positive control.

#### 2.3.3 Hemolysis and anti-haemolysis assays

The investigation into the stimulation of the extracts was conducted utilising an optimised erythrocyte hemolytic assay (Qian et al., 2011). The experimental systems contained 700 μL PBS (0.01 M, pH 7.4), 100 μL of the sample, and 200 μL of a murine erythrocyte suspension (4% v/v in PBS) that had been freshly prepared. The mixtures were then subjected to an incubation period at 37 ± 0.5 °C for 10 minutes within a thermostatic water bath, after which they were centrifugated at 12,000 × g for 5 minutes at 4 °C. Subsequently, 200 μL of the resulting mixtures were transferred to 96-well microplates for the hemoglobin quantification at 540 nm. Three replicates were performed for each sample with the following controls: 200 μL of PBS was used to replace equivalent volumes of murine erythrocyte suspension, serving as a chromogenic control; 800 μL of deionised H₂O was used to achieve complete hemolysis; and 800 μL of PBS was used to achieve null hemolysis. A serial dilution of sodium dodecyl sulfate (SDS; 1-0.0078 mg/mL in PBS) was prepared through binary dilution in order to establish the critical hemolytic concentration. Each tested sample (100 μL) was then combined with 600 μL of PBS and 200 μL of erythrocyte suspension in order to conduct an anti-haemolysis array. All procedures strictly adhered to the aforementioned hemolysis protocol.

#### 2.3.4 Hyaluronidase inhibition assay

The hyaluronidase inhibition was determined following a modified protocol (Cordero-Clavijo et al., 2025). Briefly, 100 μL of hyaluronidase solution (10 U/mL in 20 mM PBS at pH 5.35 containing 0.01 % w/v BSA and 77 mM NaCl) was mixed with 50 μL of the samples and incubated at 37 °C for 10 minutes. Subsequently, 100 μL of hyaluronic acid (0.03 % w/v in PBS at pH 5.35) was added, and the mixture was then incubated at 37 °C for 45 minutes. The reaction was then halted using 1 mL of acidic albumin, which was precipitated (10 min at room temperature) and centrifuged (12,000 × g, 5 minutes). Appropriate controls (enzyme, reagent blank, apigenin positive, vehicle) were conducted. The UV absorption of the resulting mixture was measured at 600 nm.

#### 2.3.5 Determination of the half maximal inhibitory concentration

The inhibitory rates (%) of radical scavenging capacities against DPPH• and ABTS^+^•, along with tyrosinase and hyaluronidase, were calculated using the following equation: (A_blank_–A_sample_)/A_blank_ × 100. The half-maximal inhibitory concentration (IC_50_) is defined as the analyte concentration required to achieve 50% inhibition of the target (Ma et al., 2019). The FRAP and RP experiments were conducted at an extract concentration of 0.5 mg/mL.

### 2.4 Secondary metabolomics analysis

A comprehensive metabolomic investigation was performed through the platform of Metware Biotechnology Co., Ltd., employing their validated widely targeted metabolomics workflow.

#### 2.4.1 Sample preparation

Lyophilized specimens (50.0 ± 0.5 mg) underwent a series of extractions with 1.2 mL of a 70% (v/v) methanol aqueous solution, through six cycles of vortex-assisted extraction (30 s of vortexing followed by 30 min of static incubation per cycle). Subsequently, the pooled extracts were then subjected to centrifugation at 12,000 r/min (4 °C, 3 min; D3024R centrifuge, Scilogex, USA) and filtered through 0.22 μm PTFE membranes prior to UPLC-ESI-MS/MS analysis (ExionLC™, AD system, Sciex, USA).

#### 2.4.2 Chromatographic condition

The chromatographic separation was achieved using an Agilent ZORBAX SB-C18 column (2.1 × 100 mm, 1.8 μm) maintained at 40 °C. The mobile phase was composed of solvent A (0.1% formic acid in pure water) and solvent B (0.1% formic acid in LC-MS grade acetonitrile) with a flow rate of 0.35 mL/min. The non-linear gradient elution program based on solvent B was executed as follows: 0-9.0 min: 5%-95%; 9.0-10.0 min: 95%; 10.0-11.1 min: 95%-5%; 11.1-14.0 min: 5%.

#### 2.4.3 Mass spectrometry condition

The ion source was operated in dual ESI polarity mode, with the ion spray voltage was set at 5500 V in positive ion mode and -4500 V in negative ion mode. The electrospray ionisation temperature was set at 500 °C, and the ion source gases I and II, as well as the curtain gas, were set at 50, 60, and 25 psi, respectively. The collision-induced dissociation parameter was adjusted to high.

#### 2.4.4 Metabolite annotation

Metabolite annotation was achieved using MWDB 3.0 database through orthogonal matching of retention time (± 0.2 min), exact mass (Δ <5 ppm), and MS/MS fragmentation patterns. The differential metabolites were conducted with the following thresholds: The adjusted *p*-value was less than 0.05, the |log_2_ (fold change)| was greater than 1, and the variable importance in projection (VIP) value was greater than one. The Kyoto Encyclopedia of Genes and Genomes (KEGG) pathways of the differential metabolites were constructed using the ggplot2 R package (version 3.3.0).

### 2.5 Statistical analysis

The preliminary data processing was conducted using Excel 2013. All measurements were conducted in triplicates and the results were presented as the mean ± SD. The statistical significance of differences was determined by one-way analysis of variance (ANOVA), followed by Student's t-tests for pairwise comparisons (*p* ≤ 0.05). All analyses were performed using SPSS software (version 20.0). Data visualization, including distribution diagrams, histograms, and line charts, was conducted using OriginLab 2022. Furthermore, hierarchical clustering analysis (HCA) and heatmaps were generated with TBtools, while metabolic analyses and Pearson's correlation analyses were performed using the Metware Cloud platform (https://cloud.metware.cn).

## 3 Results and discussion

### 3.1 Morphological characterization and crude extract yield of coffee beans

**Fig. 1:**
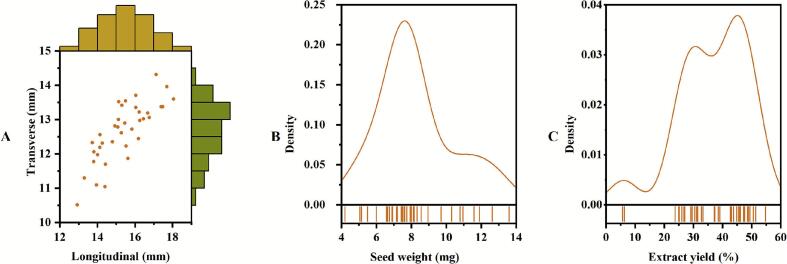
The morphological characterization, seed weight, and crude extract yield of coffee beans: (**A**) Longitudinal and transverse diameters; (**B**) Seed weight; (**C**) Extract yield.

The fruit morphology critically influences the efficiency of postharvest processing and the commercial value of agricultural commodities (Szmagara et al., 2023; Tiendrebeogo et al., 2025). In this study, the longitudinal diameter, transverse diameter, and FSI were evaluated across multiple coffee accessions (Figure 1**A**). Despite the implementation of statistical analysis, which revealed significant disparities between certain accessions, the overall variation in morphological traits was limited. The longitudinal diameter ranged from 12.93 ± 0.86 mm (‘HBN37’) to 18.06 ± 0.88 mm (‘HBN08’), with a mean of 15.38 ± 1.30 mm. The ‘HBN08’ accession was significantly longer (*p* < 0.05) than the majority of other accessions. Conversely, the transverse diameter exhibited a range from 10.52 ± 0.26 mm (‘HBN37’) to 14.31 ± 0.51 mm (‘HBN09’), with a mean of 12.65 ± 0.85 mm. ‘HBN09’ demonstrated statistically significant greater width (*p* < 0.05) in comparison to accessions bearing smaller fruits. The FSI exhibited an average value of 1.22 ± 0.06 across the various accessions, with a range from 1.11 ± 0.05 (‘HBN33’) to 1.33 ± 0.04 (‘HBN08’). ‘HBN08’ exhibited a significantly more elongated shape, whereas ‘HBN33’ was markedly more spherical than the other accessions. These extreme accessions provide valuable material for directed breeding. Despite these extreme values, the overall low coefficients of variation (longitudinal diameter: 8.46%; transverse diameter: 6.80%; FSI: 5.09%) further indicated constrained morphological diversity. These results align with previous reports (Gokavi et al., 2023; Tran et al., 2017), suggesting strong genetic stabilisation of fruit morphology in *C. arabica*, likely due to long-term selection for stable agricultural traits in this allotetraploid species (Gokavi et al., 2023).

In the context of industrial processing of plant materials, biomass and extraction efficiency of bioactive compounds represent critical economic factors (Abduh et al., 2025). In contrast to the limited morphological variation, seed-related traits exhibited substantial and statistically significant diversity. The dry seed weight varied 3.2-fold between the lightest (4.20 ± 0.85 mg in ‘HBN21’) and heaviest (13.60 ± 0.95 mg in ‘HBN31’) accessions, with a mean of 8.26 ± 2.22 mg and a high coefficient of variation (26.90%) (Figure 1**B**). Statistical analysis revealed that ‘HBN31’ exhibited significantly higher seed weight (*p* < 0.05) compared to the majority of other accessions. Furthermore, crude extract yield exhibited even greater variation, ranging from 5.67 ± 0.17% (‘HBN34’) to 54.75 ± 0.81% (‘HBN31’), with a mean of 36.87% ± 11.60% and a coefficient of variation of 31.47% (Figure 1**C**). The ‘HBN31’ accession exhibited a significantly higher (*p* < 0.05) extract yield in comparison to other accessions (except ‘HBN28’, 51.40% ± 1.32%). Comparable substantial variation in seed weight and bioactive content has been documented in other arabica coffee studies (Gokavi et al., 2023; Tran et al., 2017), possibly due to genetic disparities in metabolic pathways that influence the synthesis and accumulation of target compounds (Xue et al., 2020). These findings suggest a significant degree of plasticity in seed-related traits among coffee accessions.

The results obtained demonstrate a clear distinction in the variation patterns exhibited by morphological and seed-related traits in the context of coffee germplasm. While fruit dimensions demonstrated limited statistical differentiation, seed weight and extract yield exhibited marked and statistically significant variation, thus offering reliable criteria for genotype discrimination and breeding selection. The elite accessions such as ‘HBN02’ and ‘HBN31’, which combine high seed weight (> 10 mg) with exceptional extract yields (> 50%), represent promising targets for improving economic viability in coffee processing.

**Note:** The histograms in Figure 1**A** indicate frequency distribution of longitudinal and transverse diameters.

### 3.2 Biological activities of coffee beans

#### 3.2.1 Antioxidant activity

**Fig. 2:**
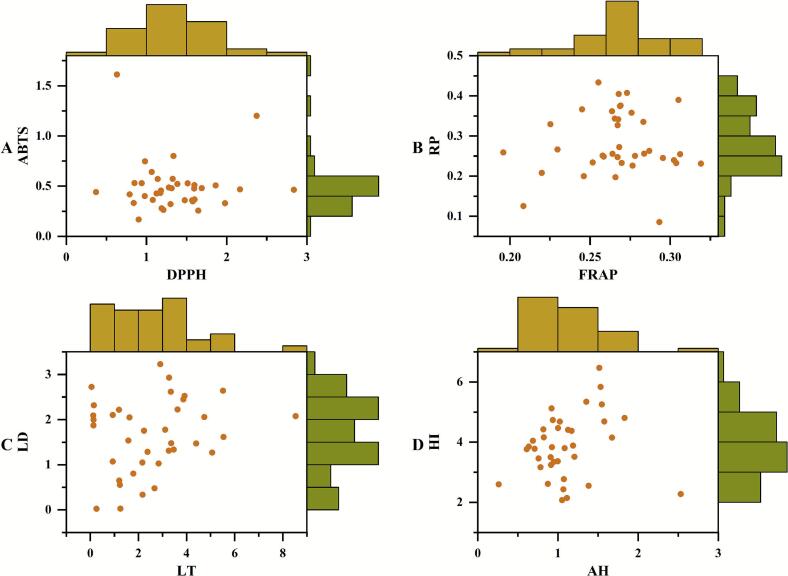
The biological activities of coffee beans: (**A**) Radical scavenging capacity; (**B**) Reducing capacity; (**C**) Tyrosinase inhibition activity; (**D**) Anti-haemolysis and hyaluronidase inhibition capacity.

The antioxidant potential of coffee beans was systematically evaluated through four complementary arrays, revealing substantial antioxidant diversity across accessions (Figure 2). The coffee beans displayed promising DPPH and ABTS^+^ radical scavenging capacities (Figure 2**A**). The mean IC_50_ value of DPPH radical scavenging capacity was 1.35 ± 0.48 mg/mL, with accession ‘HBN32’ demonstrating remarkable radical scavenging activity (0.37 mg/mL) compared to the least effective accession ‘HBN20’ (2.84 mg/mL). The mean IC_50_ value of ABTS^+^ radical scavenging capacity was 0.50 ± 0.21 mg/mL, with accession ‘HBN16’ demonstrating remarkable radical scavenging activity (0.17 mg/mL) in comparison to the least effective accession ‘HBN06 (1.61 mg/mL). Furthermore, the FRAP assay exhibited a mean value of 0.27 ± 0.03, with accession ‘HBN14’ achieving a peak value (0.32) compared to ‘HBN20’ (0.20). The mean value of the RP was 0.28 ± 0.08, with accession ‘HBN02’ achieving a peak value of 0.43 compared to ‘HBN34’ at 0.09 (Figure 2**B**). The radical scavenging assays demonstrated stronger discriminatory capability, with higher value of CV observed in DPPH (35.87%) and ABTS^+^ (51.95%) radical scavenging activities compared to FRAP (10.14%) and RP (27.90%). This divergence can be attributed to the fundamental mechanistic distinction between the two assays. Free radical scavenging assays directly quantify radical scavenging efficiency, whereas indirect reduction capacity assessments (e.g., FRAP/RP) measure reducing potential. Consequently, free radical scavenging assays are more sensitive to the redox kinetics of bioactive constituents (Zhang et al., 2023; Zhao et al., 2025). The antioxidant arrays thus established a framework for the rational selection of antioxidant accession. The elite accessions with superior DPPH and ABTS^+^ radical scavenging assays (e.g. ‘HBN09’, ‘HBN16’, ‘HBN32’ and ‘HBN35’) identified as prime candidates for functional beverages or natural antioxidants.

#### 3.2.2 Tyrosinase inhibition activity

Tyrosinase inhibition based on dual substrates (L-tyrosine and L-DOPA) demonstrated that coffee is a potential natural source of skin lightening agents (Figure 2**C**). The mean IC_50_ value of L-tyrosine-based tyrosinase inhibition was 2.57 ± 1.87 mg/mL, with accession ‘HBN19’ as the most effective accession (IC_50_ = 0.05 mg/mL) in comparison to negligible accession ‘HBN03’ (IC_50_ = 8.54 mg/mL). A parallel testing process using L-DOPA as a substrate confirmed the tyrosinase inhibition potential with mean value of IC_50_ (1.65 ± 0.81mg/mL), where accession ‘HBN24’ demonstrated near-complete inhibition (IC_50_ = 0.02 mg/mL) compared to ‘HBN22’ (IC_50_ = 3.23 mg/mL). The substantial variability (CV > 50% in both assays) positioned coffee as a natural repository for tyrosinase modulator discovery. The accession ‘HBN19’ and ‘HBN24’ with high tyrosinase inhibition demonstrated the presence of a distinctive inhibitory mechanism (Yu & Fan, 2021; Zhao et al., 2025).

#### 3.2.3 Haemolysis, anti-haemolysis, and hyaluronidase inhibition activities

Moreover, the coffee demonstrated differential anti-haemolysis (CV = 37.20%) and hyaluronidase (CV = 27.11%) inhibition activities (Figure 2**D**). The hemolysis (IC₅₀ > 10 mg/mL) of all the accessions was found to be lower than the safety standards, thereby suggesting that coffee extracts possessed a universal safety profile. The mean IC_50_ value of anti-haemolysis activity was 1.10 mg/mL, with the potent anti-irritant accession ‘HBN37’ (IC_50_ = 0.26 mg/mL) exhibiting 9.7-fold greater capacity than the least effective ‘HBN30’ (IC_50_ = 2.53 mg/mL). Furthermore, the mean IC_50_ value of hyaluronidase inhibition was 3.86 mg/mL, with the accession ‘HBN35’ exhibiting exceptional hyaluronidase inhibition capacity (IC_50_ = 2.07 mg/mL) in contrast to limited accession ‘HBN18’ (IC_50_ = 6.47 mg/mL). The elite accessions may exert their function through the inhibition of inflammatory mediators or the stabilisation of cell membranes (Cordero-Clavijo et al., 2025).

**Note:** The histograms in each figure indicate frequency distribution of activity; DPPH, 2,2-diphenyl-1-picrylhydrazyl radical scavenging activity; ABTS, diammonium 2,2’-azino-bisbis (3-ethylbenzothiazoline-6-sulfonate) radical scavenging activity; FRAP, ferric reducing antioxidant power; RP, reducing power; LT, tyrosinase inhibition activity using L-tyrosine as the substrate; LD, tyrosinase inhibition activity using L-DOPA as the substrate; AH, anti-haemolysis activity; HI, hyaluronidase inhibition activity.

#### 3.2.4 Comprehensive analysis of biological activities

**Fig. 3:**
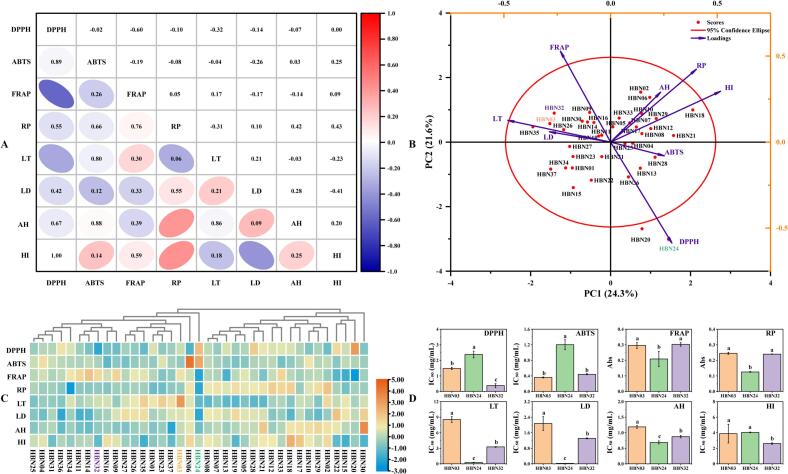
Comprehensive analysis of biological activities: (**A**) Pearson correlation analysis; (**B**) Principal component analysis; (**C**) Cluster heatmap; (**D**) Biological activities of three accessions selected.

Correlation analysis (*p* < 0.05) revealed possible relationships among antioxidant, tyrosinase inhibitory, anti-haemolysis, and hyaluronidase inhibitory parameters (Figure 3**A**). The DPPH radical scavenging capacity exhibited a significant negative correlation with FRAP (r = -0.60) and L-tyrosine-based tyrosinase inhibition (r = -0.32), while the RP exhibited significant positive correlations with anti-hemolysis (r = 0.42) and hyaluronidase inhibitory capacity (r = 0.43). The strong correlation observed between antioxidant capacity and other bioactivities is likely to be mechanistic in nature. Antioxidant components have been demonstrated to alleviate oxidative stress, a key driver of inflammation and a contributor to metabolic and neurological disorders. The scavenging of free radicals by these compounds may result in the subsequent suppression of pro-inflammatory pathways and protection against cellular damage that leads to impaired enzyme function (Qian et al., 2011; Tang et al., 2022). Furthermore, L-Dopa-based tyrosinase inhibition exhibited significant negative correlation with hyaluronidase inhibition (r = -0.41), potentially reflecting antagonistic enzymatic regulation of barrier functions (e.g., melanin synthesis vs. extracellular matrix degradation) (Cordero-Clavijo et al., 2025; Zhao et al., 2025). The majority of pairwise comparisons exhibited non-significant correlations (|r| < 0.3), thereby indicating functional orthogonality between biological activities. This independence, potentially regulated by distinct genetic or environmental factors (Aćimović et al., 2021; Lei et al., 2022), enables coordinated multi-trait improvement. The application of a correlation threshold (|r| > 0.3) facilitates strategic accession prioritisation for multifunctional ingredients, particularly formulations requiring balanced bioactivity (Aćimović et al., 2021; Grzelczyk et al., 2024). This transition from single-trait optimisation to network optimisation mirrors the intricate bioactivity interplay, as demonstrated by accession ‘HBN24’. This accession is particularly well-suited for depigmentation, a property attributable to its potent tyrosinase inhibition capability, concurrently accompanied by minimal pro-oxidant effects arising from its low antioxidant activity. Complementary accessions (e.g., the high antioxidant ‘HBN16’ and the potent anti-inflammatory ‘HBN37’) can be combined to achieve dual functionality.

Moreover, PCA (Figure 3**B**) and CHA (Figure 3**C**) further elucidated biological profile disparities among accessions, particularly for ‘HBN24’. The angular separation between DPPH and FRAP in PCA confirmed the significant negative correlation. The divergent secondary metabolite composition likely underlies differential pharmacological activities (Fallah et al., 2020; Zou et al., 2024). Consequently, metabolite profiling was focused on accession ‘HBN24’ (isolated CHA clade) and randomly selected accessions ‘HBN03’ and ‘HBN32’ (main clades) to identify metabolite composition and reveal potential active ingredients. A significant variation was observed among the three accessions in terms of DPPH, LT, LD, and AH. Conversely, the results obtained for ABTS, FRAP, and RP between ‘HBN03’ and ‘HBN32’, as well as HI between ‘HBN03’ and ‘HBN24’ exhibited non-significant differences (Figure 3**D**).

**Note:** DPPH, 2,2-diphenyl-1-picrylhydrazyl radical scavenging activity; ABTS, diammonium 2,2’-azino-bisbis (3-ethylbenzothiazoline-6-sulfonate) radical scavenging activity; FRAP, ferric reducing antioxidant power; RP, reducing power; LT, tyrosinase inhibition activity using L-tyrosine as the substrate; LD, tyrosinase inhibition activity using L-DOPA as the substrate; AH, anti-haemolysis activity; HI, hyaluronidase inhibition activity; PC, principal component; Different letters (a, b, or c) in Figure 3**D** indicate significant difference (*p* < 0.05).

### 3.3 Secondary metabolic profiles of coffee beans

#### 3.3.1 Overview of the secondary metabolism

**Fig. 4:**
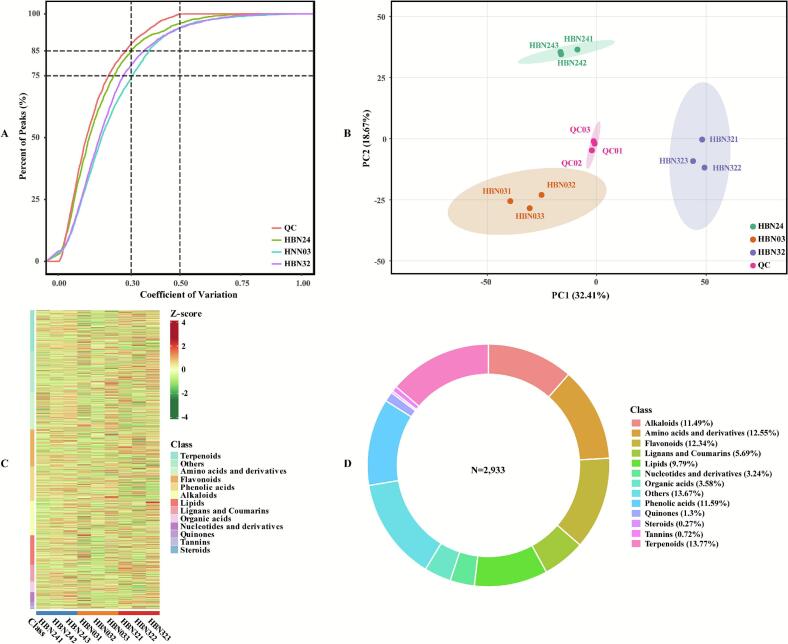
Metabolic profiles of coffee beans: (**A**) Coefficient of variation; (**B**) Principal component analysis; (**C**) Cluster heatmap; (**D**) Metabolite category.

The advent of sophisticated chromatography technology has facilitated the large-scale analysis of metabolites in numerous plant species, enabling the identification and profiling of metabolites (Deng et al., 2023; Li et al., 2022; Yin et al., 2022). The chromatographic profile concordance across multiple reaction monitoring (MRM) and total ion chromatogram (TIC) of quality control (QC) samples (**Figure S1**), with 30% of intra-batch coefficient of variation (CV) for more than 85% of detected features (Figure 4**A**), confirmed methodological stability and reliability in metabolite extraction and detection workflows (Zou et al., 2024). In order to facilitate the visualisation of the metabolite profiles for the three samples, the metabolomic data was subjected to PCA (Figure 4**B**) and HCA (Figure 4**C**). The resultant visualisation result indicated a high degree of similarity in the metabolite expression patterns within triplicate biological replicates, and a marked difference between the accessions ‘HBN03’, ‘HBN24’, and ‘HBN32’. Consequently, the result confirmed the methodological robustness of secondary metabolomic profiling in the experimental specimens and substantial heterogeneity in secondary metabolite composition across sample groups.

A total of 2,933 structurally defined metabolites were identified in positive and negative ion modes (**Table S2**). The present study identified a greater number of metabolites than was previously reported (Hong et al., 2025; Pimenta et al., 2025; Wu et al., 2025), thus demonstrating the efficacy of widely targeted metabolomics for comprehensive metabolite profiling in coffee beans. These metabolites were divided into 13 major categories (Figure 4**D**). The top five classes were terpenoids (13.77%), amino acids and derivatives (12.55%), and flavonoids (12.34%), phenolic acids (11.59%), and alkaloids (11.49%), constituting the core profile with balanced proportions. Among these, amino acids and their derivatives reflect active primary metabolites (Bichlmaier et al., 2025; Wu et al., 2025), while the dominant terpenoids, flavonoids, phenolic acids, and alkaloids (accounting for approximately 50% of the total) indicate the robust secondary metabolic capacity (Hong et al., 2025; Pimenta et al., 2025; Santanatoglia et al., 2024). These secondary metabolites have been found closely associated with key physiological and ecological functions, including antioxidant activity, stress resistance, signal transduction, and pigment formation (Ben ElHadj Ali et al., 2020; Ma et al., 2019; Oh et al., 2023). This metabolic profile provides a valuable foundation to reveal potential constituents that may be responsible for the observed differences in biological activity among the three accessions.

**Note:** QC, quality control; PC, principal component.

#### 3.3.2 K-means analysis of metabolites

**Fig. 5:**
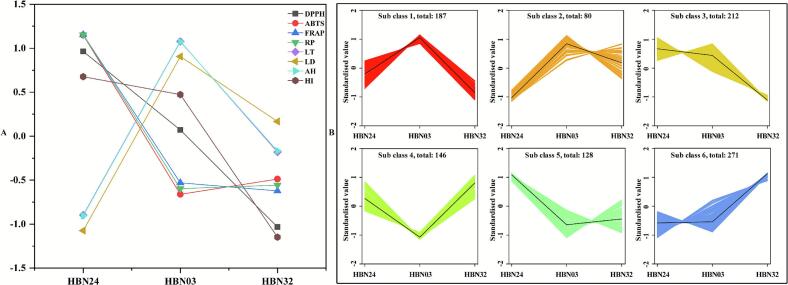
K-means analysis: (**A**) Biological activities; (**B**) Metabolites.

**Note:** DPPH, 2,2-diphenyl-1-picrylhydrazyl radical scavenging activity; ABTS, diammonium 2,2’-azino-bisbis (3-ethylbenzothiazoline-6-sulfonate) radical scavenging activity; FRAP, ferric reducing antioxidant power; RP, reducing power; LT,

The K-means clustering of metabolite abundance across the three accessions systematically characterised metabolic variation. Elbow analysis determined six optimal metabolic groups (Figure 5). Cluster 2 exhibited the strongest correlation with ABTS, FRAP, and RP, while Cluster 5 demonstrated the strongest correlation with LT, LD, and AH. Furthermore, Cluster 6 showed the strongest correlation with DPPH and HI, as evidenced by co-varying trends between metabolite abundance and results. Within Cluster 2, 80 metabolites were classified into 12 subclasses, dominated by flavonoids (n = 20) and terpenoids (n = 16), with nobiletin (flavonoid) and geniposidic acid (terpenoid) as the most abundant compounds (**Table S2**). Within cluster 5, 128 metabolites were classified into 12 subclasses, dominated by flavonoids (n = 29). Catechin-(7,8-bc)-4-(3,4-dihydroxyphenyl)-dihydro-2-(3H)-one showed the highest abundance. In cluster 6, 271 metabolites were classified into 12 subclasses, dominated by flavonoids (n = 61). Of particular note flavonoid was syringetin-3-O-glucoside. In summary, the compositional divergence in flavonoids across clusters likely underpins the differential bioactivities observed.

L-tyrosine-based tyrosinase inhibition; LD, L-DOPA-based tyrosinase inhibition; AH, anti-haemolysis activity; HI, hyaluronidase inhibition activity.

#### 3.3.3 Key differential metabolite analysis of coffee beans between three accessions

In the three comparative analyses under consideration, the samples were found to be well within the confidence interval, with R^2^ Y and Q^2^ values exceeding 0.89. The reliability of the model was confirmed by the steep slopes of the lines from the 200-fold permutation test, indicating the goodness of fit of the model. The top 20 differential metabolites, ranked by VIP scores, are listed in Supplementary **Table S3**, highlighting key classes such as terpenoids, phenolic acids, and flavonoids.

**(1) Differential metabolites between HBN03 and HBN24**

**Fig. 6:**
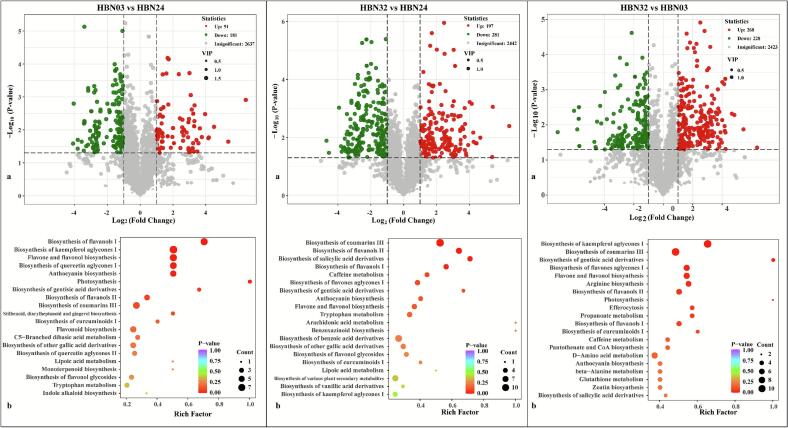
Determination and enriched metabolic pathways of differential metabolites: (**a**) volcano plot; (**b**) TOP20 enriched metabolic pathways.

A total of 272 differential metabolites were identified between HBN03 and HBN24 (Figure 6**A**), dominated by flavonoids (55), terpenoids (53), and phenolic acids (28). Among them, 91 upregulated metabolites in HBN03 may contribute to the high antioxidant activity, and 181 upregulated metabolites in HBN24 may contribute to the high tyrosinase inhibition and anti-hemolysis activity.

**(2) Differential metabolites between HBN32 and HBN24**

A total of 478 differential metabolites were identified between HBN32 and HBN24 (Figure 6**B**), dominated by terpenoids (97), flavonoids (83), phenolic acids (73), alkaloids (48), and amino acids and derivatives (47). Among them, 197 upregulated metabolites in HBN32 may contribute to higher antioxidant and hyaluronidase inhibition activity, and 281 upregulated metabolites in HBN24 may contribute to higher tyrosinase inhibition and anti-haemolysis activity.

**(3) Differential metabolites between HBN32 and HBN03**

A total of 496 differential metabolites were identified between HBN32 and HBN03 (Figure 6**C**), including 89 flavonoids, 71 terpenoids, 65 phenolic acids, 59 amino acids and derivatives, 54 alkaloids, 36 lignans and coumarins, 36 lipids, 16 organic acids, 15 nucleotides and derivatives, 6 quinones, 3 tannins, 1 steroid, and 45 other substances. Among them, 268 upregulated metabolites in HBN32 may contribute to higher DPPH radical scavenging capacity, tyrosinase and hyaluronidase inhibition, and anti-haemolysis activity.

#### 3.3.4 The main enriched metabolic pathways of differential metabolites

The TOP20 enrichment metabolic pathways of differential metabolites among three coffee beans were filtered out for further analysis. In comparison with HBN24, a total of 90 differential metabolites in HBN03 were annotated and significantly enriched in several flavonoid-related pathways, including biosynthesis of flavanols I, biosynthesis of kaempferol aglycones I, flavone and flavonol biosynthesis, biosynthesis of quercetin aglycones I, and anthocyanin biosynthesis (*P*-value < 0.01). These pathways are closely associated with the biosynthesis of key polyphenolic compounds that influence coffee’s antioxidant capacity and sensory quality (Santanatoglia et al., 2024). It was found that 128 differential metabolites in HBN32 were enriched in the pathways, such as biosynthesis of coumarins III, biosynthesis of flavanols II, and biosynthesis of salicylic acid derivatives. Coumarins are known to contribute to bitterness and aroma (Ma et al., 2019; Zhang et al., 2023), while salicylic acid derivatives may play a role in flavor modulation (Wu et al., 2025). Furthermore, a total of 153 differential metabolites between HBN03 and HBN32 were annotated and enriched in the pathways of biosynthesis of kaempferol aglycones I, biosynthesis of coumarins III, and biosynthesis of gentisic acid derivatives. In general, the results indicated significant involvement of flavonoid and organic acid metabolic pathways in differentiating the metabolic profiles among coffee accessions, suggesting their potential roles in shaping flavor characteristics and bioactivities in coffee beans.

**Note**: VIP, variable importance in projection

By correlating metabolites with bioactivities, we have preliminarily identified potential constituents responsible for observed variations in biological activity. Notably, the differential flavonoid component syringetin-3-O-glucoside and the signature organic acid chlorogenic acid have been the focus of significant research in the field of coffee studies (Grzelczyk et al., 2024; Han et al., 2024; Pimenta et al., 2025). Specific metabolite classes were confirmed as key contributors to bioactivity. Flavonoids, terpenoids, and organic acids, abundant in variety and diverse in structure, constitute the main bioactive components in coffee beans contributing to health benefits, while amino acids type and content serve as key indicators for evaluating nutritional and flavour properties (Bichlmaier et al., 2025; Grzelczyk et al., 2024; Hong et al., 2025; Santanatoglia et al., 2024). The observed bioactivities are likely not attributable to a single compound but rather arise from synergistic or additive effects within the complex coffee matrix. Furthermore, the levels of these nutritional and health-promoting components are significantly influenced by geographic origin, roasting methods, digestion, and brewing processes (Bichlmaier et al., 2025; Grzelczyk et al., 2024; Pimenta et al., 2025; Santanatoglia et al., 2024). It is important to note that widely targeted metabolomics has limitations for absolute quantification and quality evaluation, but the relative contents of identified components are highly relevant to potential health benefits (Deng et al., 2023; Yin et al., 2022; Zou et al., 2020). Consequently, future research should prioritise absolute quantification of targeted components based on these results and consider synergistic or additive effects within the complex, thereby enabling precise evaluation of health benefits.

## 4 Conclusion

This systematic investigation of 37 coffee accessions establishes a clear link between their metabolite profiles and bioactivities. Three distinct accessions (HBN03, HBN24, HBN32) and a comprehensive set of 2,933 metabolites were identified. Flavonoids and organic acids were found to be the most abundant classes correlated with bioactivity. The application of K-means and correlation analysis, with particular emphasis on compounds such as like chlorogenic acid, provides a predictive framework for the rational selection of coffee accessions. Consequently, this study provides a metabolomic foundation for the development of coffee-based functional foods and cosmeceuticals, with the identified accessions representing promising candidates for further product development.

## CRediT authorship contribution statement

**Yuan Wang:** Writing – original draft, Visualization, Formal analysis. **Zizheng Huang:** Methodology, Investigation, Conceptualization. **Hongyan He:** Investigation, Formal analysis. **Xiandong Zhou:** Validation, Investigation. **Dingshan Yang:** Validation, Investigation. **Peng Shu:** Supervision, Data curation. **Xueqing Chen:** Writing – review & editing, Project administration, Funding acquisition, Conceptualization. **Jinhuan Cheng:** Writing – review & editing, Resources. **Yao Ma:** Writing – review & editing, Project administration, Funding acquisition, Conceptualization.

## Declaration of Competing Interest

The authors declare that they have no known competing financial interests or personal relationships that could have appeared to influence the work reported in this paper.

## Data Availability

Data will be made available on request.

## Appendix A Supplementary data

Chromatographic profile Image 1

Sample information and defined metabolites Image 2
