# Supplementary material for: Phytoconstituents, antioxidant and enzyme inhibition activities of coffee beans through widely targeted metabolomes and in vitro arrays
Source: Food Chem X. 2025 Nov 10;32:103280. doi: 10.1016/j.fochx.2025.103280 (PMC12657746; doi:10.1016/j.fochx.2025.103280)
Supplement: Supplementary file 1 — Chromatographic profile [file mmc1.docx]

**Supplementary** **Figures**


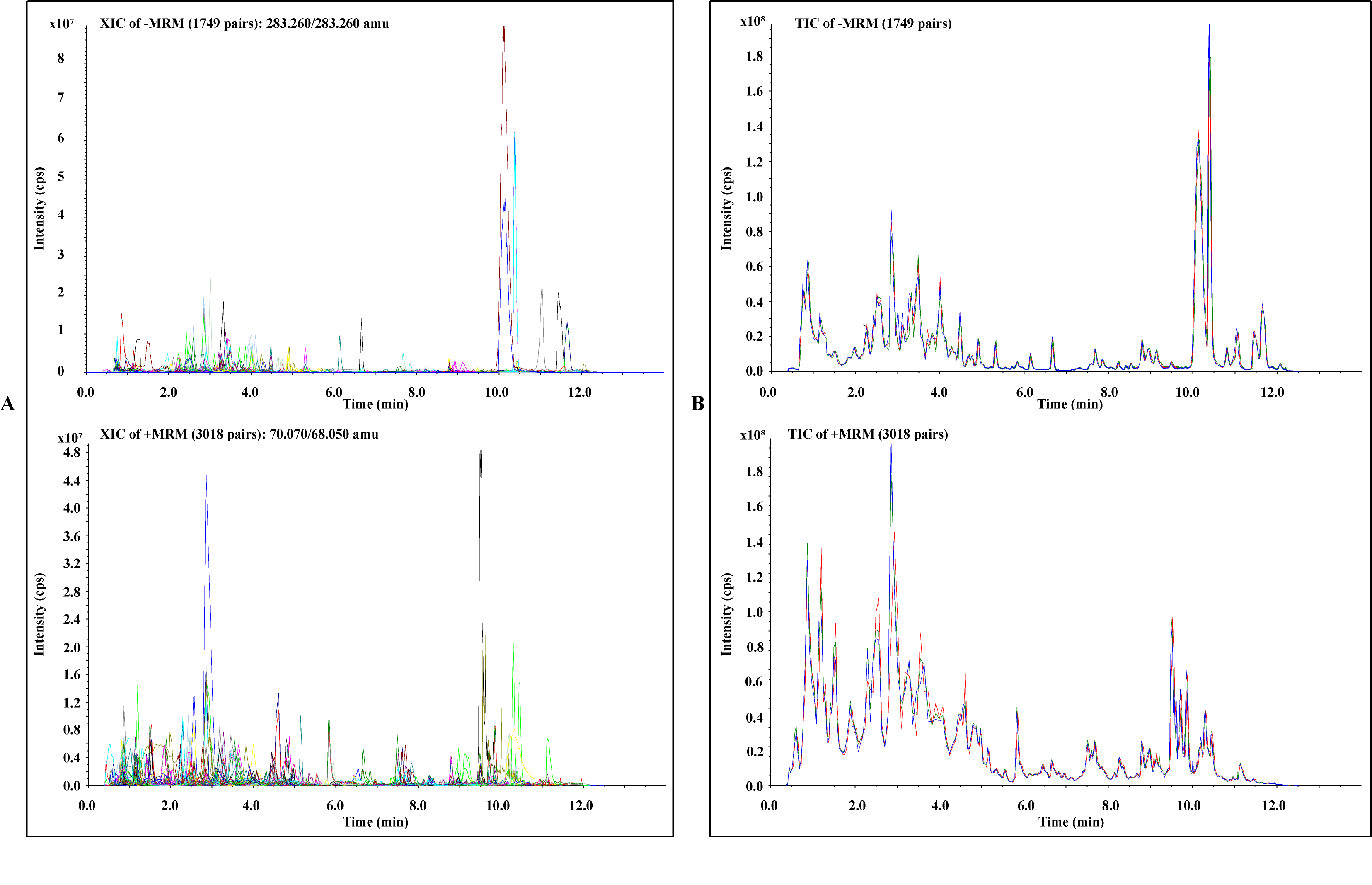


**Figure S1** Chromatographic profile concordance across multiple reaction monitoring (MRM) and total ion chromatogram (TIC) of quality control samples
